# Downregulation of Cellular Protective Factors of Rumen Epithelium in Goats Fed High Energy Diet

**DOI:** 10.1371/journal.pone.0081602

**Published:** 2013-12-09

**Authors:** Manfred Hollmann, Ingrid Miller, Karin Hummel, Sonja Sabitzer, Barbara U. Metzler-Zebeli, Ebrahim Razzazi-Fazeli, Qendrim Zebeli

**Affiliations:** 1 Department for Farm Animals and Veterinary Public Health, Institute of Animal Nutrition and Functional Plant Compounds, Vetmeduni Vienna, Vienna, Austria; 2 Department of Biomedical Sciences, Institute of Medical Biochemistry, Vetmeduni Vienna, Vienna, Austria; 3 VetCore Facility for Research, Vetmeduni Vienna, Vienna, Austria; 4 Department for Farm Animals and Veterinary Public Health, Clinic for Swine, Vetmeduni Vienna, Vienna, Austria; University of S. Florida College of Medicine, United States of America

## Abstract

Energy-rich diets can challenge metabolic and protective functions of the rumen epithelial cells, but the underlying factors are unclear. This study sought to evaluate proteomic changes of the rumen epithelium in goats fed a low, medium, or high energy diet. Expression of protein changes were compared by two-dimensional differential gel electrophoresis followed by protein identification with matrix assisted laser desorption ionisation tandem time-of-flight mass spectrometry. Of about 2,000 spots commonly detected in all gels, 64 spots were significantly regulated, which were traced back to 24 unique proteins. Interestingly, the expression profiles of several chaperone proteins with important cellular protective functions such as heat shock cognate 71 kDa protein, peroxiredoxin-6, serpin H1, protein disulfide-isomerase, and selenium-binding protein were collectively downregulated in response to high dietary energy supply. Similar regulation patterns were obtained for some other proteins involved in transport or metabolic functions. In contrast, metabolic enzymes like retinal dehydrogenase 1 and ATP synthase subunit beta, mitochondrial precursor were upregulated in response to high energy diet. Lower expressions of chaperone proteins in the rumen epithelial cells in response to high energy supply may suggest that these cells were less protected against the potentially harmful rumen toxic compounds, which might have consequences for rumen and systemic health. Our findings also suggest that energy-rich diets and the resulting acidotic insult may render rumen epithelial cells more vulnerable to cellular damage by attenuating their cell defense system, hence facilitating the impairment of rumen barrier function, typically observed in energy-rich fed ruminants.

## Introduction

Rumen is a classical host-microbial ecosystem that enables ruminant animals the conversion of largely indigestible plant biomass into valuable food products, making them highly significant to human nutrition. Complex dietary carbohydrates are degraded in the rumen by microbial glycosidases to short-chain fatty acids, which are absorbed and subsequently metabolized in the rumen epithelium, supplying energy to the host [1]. Besides their central metabolic roles, rumen epithelial cells also are the first line of defence against hostile rumen conditions such as acidic pH, high osmotic pressure, and harmful microbial-derived metabolites, in particular when ruminants are fed energy-rich diets to enhance cost efficiency [2]. Indeed, a direct implication of feeding energy-rich diets to ruminants is rumen acidosis, resulting is an enhanced impairment of barrier functions of the rumen epithelium [3], [4], inflammation and other health-related problems of the host animal [5]. The wide use of high-throughput molecular methods in animal sciences during the last years has increased the knowledge regarding rumen microbiota and its interaction with the host in response to diet [2], [6]–[10]. However, there is a paucity of information with regard to the underlying mechanisms behind the disruption of protective functions of rumen epithelium and its metabolic consequences in response to energy-rich diets.

Development of efficient prophylactic feeding strategies against rumen disorders requires a system-wide understanding of metabolic and protective functions of the rumen epithelium. Proteomic technologies enable simultaneous analyses of thousands of proteins in a tissue, therefore providing valuable tools for in-depth research in farm animals and nutritional sciences [11]. To our best knowledge, only one study used a proteomic approach to investigate changes in the rumen epithelium in sheep [8], whereas no information exists about rumen epithelium of other ruminant species including goats.

The main aim of this study was to investigate changes in the protein expression of rumen epithelium in response to energy-rich diets in goats. Protein pattern differences were detected by sensitive two-dimensional differential gel electrophoresis (2D-DIGE) in combination with matrix assisted laser desorption ionisation tandem time-of-flight (MALDI-TOF/TOF) mass spectrometry (MS) for protein identification. These results were complemented by determination of mRNA expression of the corresponding genes to evaluate transcriptional changes. Together, results from this investigation provide novel insights into proteomic changes of rumen epithelium of goats, identifying numerous hitherto unknown potential protective factors that were downregulated in the rumen epithelium by high dietary energy supply.

## Materials and Methods

### Ethics Statement

The animal experimentation protocol of this research was approved by the institutional ethics committee of the University of Veterinary Medicine Vienna in accordance with Good Scientific Practice guidelines and national legislation (Protocol no. 15/03/97/2011).

### Animals and Study Design

Animals of this study were used in an Ussing-chamber experiment to evaluate the effects of energy-rich feeding on the electrophysiological properties of rumen epithelium [4], as well as to determine nutrient transporters and electrochemical gradients in rumen and hindgut [12]. The outline of the experimental design, feeding procedure, and dietary treatments are therefore the same as has been described in details previously [4], [12]. In brief, 18 growing goat kids, 4 months of age, were used. Animals were arranged to three dietary treatment groups consisting of low, intermediate, or high energy supply. The low diet was composed purely of hay, and was considered a control diet, whereas the medium and high energy diets consisted in hay plus 30 and 60% barley grain in dry matter (DM) basis, respectively. The hay used was a second cut chopped meadow hay containing 88.0% DM, 11.2% crude protein (CP), 91.6% organic matter (OM), 55.6% neutral detergent fiber (NDF) and 8.8 MJ metabolizable energy (ME) pro each kg DM, whereas barley grain was coarsely ground with 88.3% DM, 96.9% OM, 13% CP, 20.5% NDF, and 13.0 MJ ME pro kg DM. The feed amount was calculated to meet energy and nutrient requirements for maintenance and to account for 250 g body weight (BW) gain per wk in animal fed the low diet [13]. During the experiment, goats had free access to water and mineral lick stone (Alpenleckmasse für Rinder: Ca, 12%; P, 6%; Na, 5%; Mg, 2%; Zn, 6,000 mg; Mn, 2,000 mg; Cu, 1,000 mg; I, 40 mg; Se, 40 mg; GARANT Tiernahrung, Pöchlarn, Austria) and a salt lick stone (Biosaxon Salzleckstein, sodium chloride, Na min. 39%; GARANT Tiernahrung, Pöchlarn, Austria). Goats were clinically healthy throughout the experiment and no animal was in need of veterinary treatment except of one goat assigned to the low group that died before starting the experiment. Thus, only 5 animals were in the low energy group, whereas the other two groups consisted of 6 animals each.

### Sample Collection and Preparation of Rumen Tissues

Goats were euthanized 2 to 3 hours after the last morning feeding with an intravenous injection of 50 mg/kg BW Thiopental (Thiopental Sandoz 1 g - Trockenstechampulle, Sandoz, Austria) after sedation with intravenous injection of 0.2 mg/kg BW Xylazin (Xylasol Injektionslösung für Tiere 50 ml; Ogris Pharma, Wels, Austria). The abdominal cavity was immediately opened by midline incision and the rumen was carefully removed. The rumen was opened from the dorsal side. The pH was measured in the rumen fluid using an electrode connected to a pH-meter (Seven Multi TM, Mettler-Toledo GmbH, Schwerzenbach, Switzerland). Approximately 5×5 cm of rumen wall tissue was excised from the cranial part of the ventral sac and cleaned in phosphate-buffered saline (PBS) buffer. The rumen epithelium was separated from the muscular and serosal layers and cut into small pieces (approximately 0.5 cm^2^), snap-frozen in liquid nitrogen, and stored at −80°C. The same set of rumen tissue samples was used for mRNA and protein expression. The frozen rumen tissue samples were ground thoroughly to a fine powder with mortar and pestle in liquid nitrogen. A subsample (80 mg) was dissolved in 300 µl DIGE lysis buffer (8 M Urea, 4% CHAPS, 30 mM Tris-HCl, pH 8.5), kept overnight at 4°C and used for 2D-DIGE analysis. The tissue extract was centrifuged (20 minutes at 30,000× g, 4°C), and the supernatant containing the solubilised proteins was stored at −80°C until analyzed. Protein concentration was determined using a commercial Bradford reagent (Bio-Rad, Hercules, CA).

### 2D-DIGE Analysis

The 2D-DIGE experiment was designed to compare samples of all three treatments. Specimens were pre-electrophoretically labelled with the Refraction-2D labelling kit (NH DyeAGNOSTICS, Halle, Germany) according to [14] and [15]. A pool of equal amounts of protein from all samples was used as internal standard. Two samples (labelled with G-Dye 200 and G-Dye 300, respectively) and the internal standard (labelled with G-Dye 100) were separated on each gel, with a protein load of 50 µg per specimen and dye. Labelled and pooled proteins were loaded on IPG strips (3–10 pH range, nonlinear, 17 cm; Bio-Rad) by active rehydration for 8 h at 50 V and then separated in a Protean IEF cell (Bio-Rad) by stepwise increasing voltage for a total of 45 000 Vh. Subsequently, strips were equilibrated first for 15 min in 50 mM Tris-HCl (pH 8.8), 6 M urea, 2% SDS, 20% glycerol, 130 mM dithiothreitol (DTT), then for 15 min in the same solution but containing 135 mM iodoacetamide instead of DTT. The second-dimensional separation was performed in 12% homogeneous SDS polyacrylamide gels in a Protean II xi Cell (Bio-Rad) at a constant current of 10 mA/gel over night.

### Image Acquisition and Analysis

All DIGE-gels were scanned with a Typhoon 9400 variable mode imager (GE Healthcare Life Sciences, Munich, Germany) immediately after the run at appropriate wavelengths [14], [15]. Spot patterns were evaluated by using DeCyder software (version 7.0, GE Healthcare). Ratios between volumes of single spots in the samples and the corresponding spots in the internal standard were calculated, and significance for regulated spots was determined, using the statistical features in DeCyder. Protein spots differentially expressed between groups were extracted, using volume ratios (change of at least 30% in one of the comparisons) and Student's t-test (*P*≤0.05) as selection criteria.

### Mass Spectrometry using MALDI-TOF/TOF and Data Analysis

After scanning, 2D-DIGE gels were silver stained [15]. Differentially regulated spots were located and excised manually from gels, washed, destained [16], reduced with DTT and alkylated with iodoacetamide [17]. All washing steps before and after destaining as well as after reduction/alkylation were performed with 100 mM aqueous ammonium bicarbonate.

In-gel digest proceeded for 8 hours at 37°C with trypsin (Trypsin Gold, Mass Spectrometry Grade, Promega, Madison, WI) according to [18] with a final trypsin concentration of 20 ng/µl in 50 mM aqueous ammonium bicarbonate and 5 mM CaCl_2_. Afterwards, peptides were extracted with three changes of 30 µL of 5% trifluoro acidic acid in 50% aqueous acetonitrile supported by ultrasonication for 10 min per change. Extracted peptides were dried down in a vacuum concentrator (Eppendorf, Hamburg, Germany).

Dried peptides were concentrated and de-salted using Zip-Tips C18 (microbed) (Millipore, Billerica, MA) according to the manufacturer's instructions. De-salted peptides (0.5 µl) were spotted onto a disposable AnchorChip MALDI target plate pre-spotted with α-cyano-4-hydroxycinnamic acid (PAC target, Bruker Daltonics, Bremen, Germany). Data were acquired on a MALDI-TOF/TOF mass spectrometer (Ultraflex II, Bruker Daltonics, Bremen, Germany) in MS and MS/MS modes. Spectra processing and peak annotation were carried out using FlexAnalysis and Biotools (both Bruker Daltonics, Bremen, Germany) as described previously [19].

For database searches, the processed spectra were searched via an in-house Mascot server (Matrix Science, Boston, MA) and the software ProteinScape (Bruker Daltonics, Bremen, Germany) in the Swiss-Prot database or in NCBInr (National Center for Biotechnology Information, http://www.ncbi.nlm.nih.gov/) using the following search parameters: taxonomy mammalia; global modifications carbamidomethylation on cysteine; variable modifications oxidation on methionine; deamidation on asparagine and glutamine as well as formation of pyroglutamic acid; MS tolerance 100 ppm; MS/MS tolerance 1 Da; one missed cleavage allowed.

### Quantitative qPCR

Samples were processed as previously described [12] and the high quality RNA samples were reused in this study. The RNA quality was determined with On-Chip-Electrophoresis (Agilent Technologies, St. Clara, CA) [12]. Quantitative real-time RT-PCR was used to estimate the relative mRNA expression levels of the selected genes of interest [12]. Primers (Sigma-Aldrich, St. Louis, MO) used for amplification of target genes (Table S1) were designed using Primer Express Software version 2.0 or 3.0 (Applied Biosystems, Foster City, CA). Homologous sequences from cattle, sheep and goat were aligned in order to find conserved regions. Primers were located on different exons which combined with the DNAse I treatment of the samples should prevent the amplification of contaminating DNA. Reverse transcription controls (RT minus) were included in order to screen for residual DNA contamination. Synthesis of cDNA was performed using the utilizing High Capacity Reverse Transcription Kit (Life Technologies, Carlsbad, CA) by following the manufacturer's protocol. Two µg total RNA per sample were used as starting material for reverse transcription. Quantitative PCR was performed on the StepOne Plus Real-Time PCR system (Applied Biosystems) using the Power SYBR Green PCR Master Mix (Life Technologies) in 20 µl reactions mixes including 150 nM of each primer and 2 µl of cDNA template (diluted 1∶4). A standard curve was included as an internal reference in each measurement to exclude technical variability. The reaction efficiency of all assays was determined and is provided in Table S1. Afterwards the length of the RT - qPCR product was analysed by a running gel. All samples were analysed in duplicates. In order to detect non-specific amplification and primer-dimer formation a melting curve profile was generated at the end of each run. One specific gene was detected in each sample. The following temperature profile was used: The initial activation step (10 min at 95°C) was followed by 40 cycles with 15 s of denaturation at 95°C, annealing and extension for 1 min at 60°C. The melting curve was analysed using an initial denaturing step at 95°C for 15 s, cooling to 60°C for 1 min and then increasing the temperature to 95°C at a ramping rate of 0.3°C/s. Relative gene expression levels were calculated using the ΔΔCt method [20]. This method contains a normalisation and calibration step. The low energy goat group was used as control group for calibration. After normalisation a defined animal was determined as calibrator and set to 1. The calibration step was performed for each gene separately. The expression levels of the target genes were normalized to those of the two most suitable housekeeping genes, namely ACTB/HPRT1 [12]. Four housekeeping genes (ACTB, OAZ1, HPRT, and GAPDH) were proved with gNorm [21] and evaluated by stability ranking with stability values ranging from 0.20 to 0.45.

Gene expression data were analyzed as completely randomized design using the mixed-model procedure of SAS (SAS Inst. Inc., Cary, NC) with goat as random effect and diet being the fixed effect. Degrees of freedom were approximated using Kenward-Rogers method (ddfm  =  kr). Multiple comparisons were done with the PDIFF option of SAS. Means were reported as least-squares means ± pooled standard error of the means (SEM) with *P*≤0.05 defined as significant.

## Results

Average daily DM intakes of goats at the end of experiment were 705±72, 708±16, and 883±19 g DM/d, resulting in an average energy intake of 6.2, 7.3, and 10.2 MJ ME/animal/d for low, medium, and high groups, respectively. Ruminal pH, measured in the rumen fluid at the same time of mucosal sample collections, was lower in the high group (5.5) than in low group (6.4) (*P*<0.001), whereas the pH values of low vs. medium group (6.0), and medium vs. high group did not differ (*P*>0.05) between them.

### Impact of Dietary Energy Supply on Proteomic Changes

To evaluate the effects of diet-induced acidotic challenge, a comparative proteomic analysis was performed using the highly sensitive 2D-DIGE approach. The overall number of protein spots separated by this technique was approximately 2,600 from each tissue sample. From around 2,000 protein spots detected, 64 spots were differently regulated (Table 1), whereby at least 75% of all gels had a 1.25-fold change in the abundance ratio (Student's t-test, *P*<0.05). Data showed that 12 spots were differentially expressed between groups of goats fed low vs. medium diet and other 12 spots differed between medium and high energy diets (Table 1). Interestingly, the analysis indicated that 40 protein spots were differentially regulated in the rumen epithelium between goats fed low vs. high energy diets, whereby 33 of these spots were downregulated and 7 were upregulated when animals were shifted from low to high energy diet (Table 1).

**Table 1:** Summary of differentially expressed and identified spots from 2D-DIGE gels of rumen epithelium of goats fed diets differing in the energy supply.

Compared diets^1^	Regulated spots^2^	Up-regulated spots^2^	Down-regulated spots^2^	Identified spots
L vs. M	12	11	1	2
M vs. H	12	3	9	6
L vs. H	40	7	33	22

1 L  =  low energy diet; M  =  medium energy diet, H  =  high energy diet.

2 At least 30% difference in expression.

**Figure 1:**
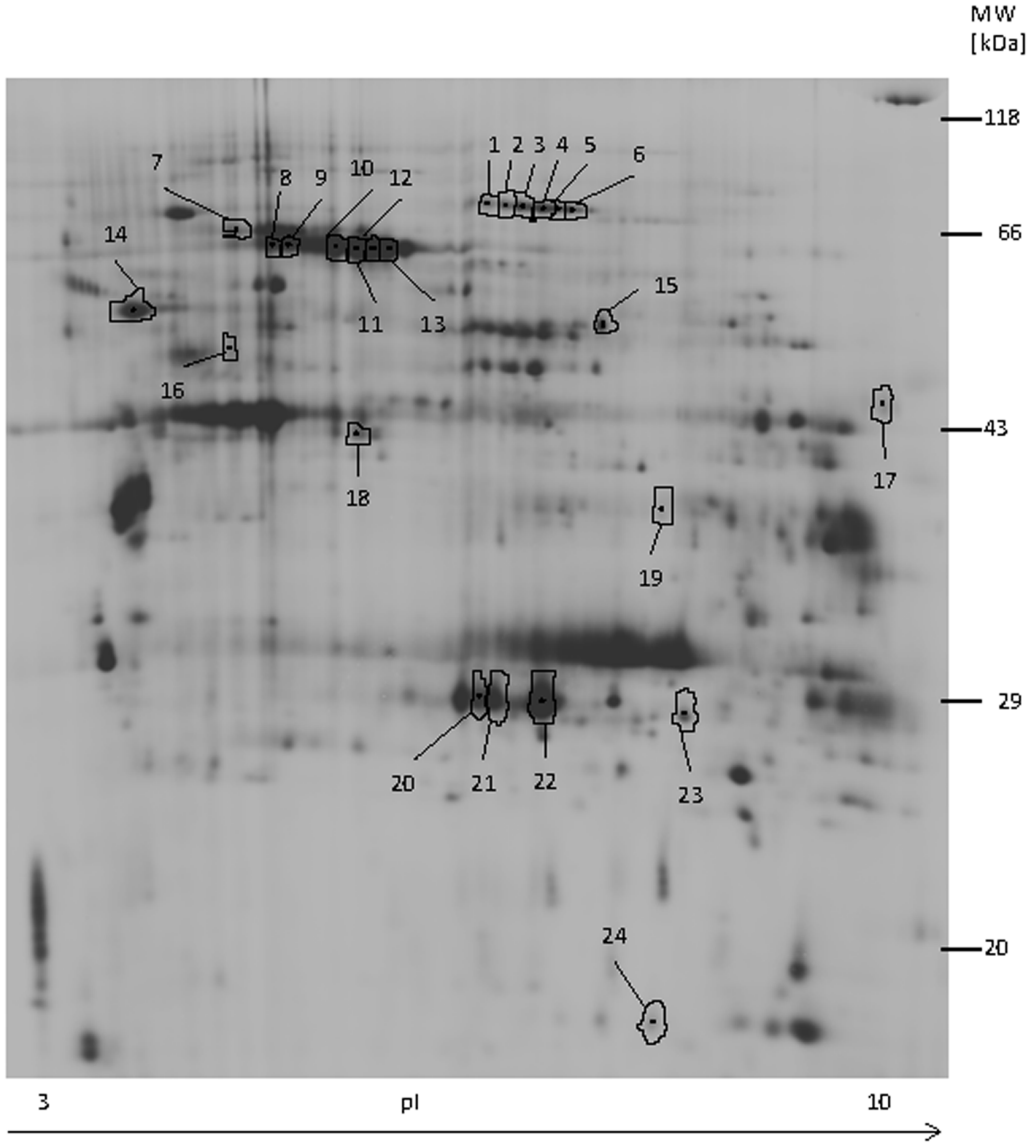
Representative 2DE-DIGE gel image of differentially expressed proteins after silver staining. Numbers correspond to the identified proteins in  2.

For protein identification in MALDI-TOF/TOF-MS, 40 spots were selected based on the following quality criteria: expression pattern in the 2D-DIGE analysis (i.e., the extent of difference between the dietary treatment groups), stainability of spots with silver for colorimetric visualisation on the gels enabling manual spot cutting, spot density in silverstained patterns supplying sufficient amounts for MS analysis and spot quality (i.e., the shape and compactness of spots, not overlapping with others). Using this approach and data-based research, 24 out of the 40 selected protein spots were successfully identified; they are marked in Figure 1 which shows a representative 2D-DIGE gel post-stained with silver. The identified spots and the corresponding protein names are listed in Table 2 including their accession number, p*I*, theoretical mass, sequence coverage, and average fold changes. A total of 12 proteins were identified, and among them 3 proteins were found in multiple spots, such as transferrin (identified in spots 1 to 6), albumin precursor (spots 8 to 13), and peroxiredoxin-6 (PRDX6) (identified in spots 20 to 22) (Figure 1; Table 2).

**Table 2:** Differentially expressed proteins in the rumen epithelium of goats fed diets differing in the energy supply^1^.

						Av. Ratio		
Spot^2^	Theoretical mass (kDa)	pI	SC^3^ (%)	Accession^4^	Annotation	L vs. M	M vs. H	L vs. H
1	6.6^5^	9.5	39.3	gi|2318026	Transferrin	−1.18	−1.30	−1.53**
2	6.6^5^	9.5	39.3	gi|2318026	Transferrin	−1.22	−1.39	−1.69**
3	6.6^5^	9.5	39.3	gi|2318026	Transferrin	−1.23	−1.46	−1.79**
4	6.6^5^	9.5	39.3	gi|2318026	Transferrin	−1.24	−1.24	−1.54*
5	6.6^5^	9.5	39.3	gi|2318026	Transferrin	−1.28	−1.42	−1.82**
6	6.6^5^	9.5	39.3	gi|2318026	Transferrin	−1.33	−1.54	−2.06**
7	71.2	5.2	9.2	HSP7C_BOVIN	Heat shock cognate 71 kDa protein	−1.04	−1.26*	−1.31*
8	66.3	5.5	6.5	gi|193085052	Albumin precursor	−1.15	−1.16	−1.33*
9	66.3	5.5	9.3	gi|193085052	Albumin precursor	−1.41	−1.48	−2.09**
10	66.3	5.5	9.6	gi|193085052	Albumin precursor	−1.47*	−1.31	−1.92**
11	66.3	5.5	6.5	gi|193085052	Albumin precursor	−1.41	−1.36	−1.92*
12	66.3	5.5	7.2	gi|193085052	Albumin precursor	−1.52	−1.38	−2.10**
13	66.3	5.5	9.1	gi|193085052	Albumin precursor	−1.57	−1.22	−1.91*
14	57.2	4.7	6.3	PDIA1_BOVIN	Protein disulfide-isomerase	+1.48*	−1.82*	−1.23
15	54.8	6.4	11.4	ALIA1-SHEEP	Retinal dehydrogenase 1	+1.38	+1.13	+1.56*
16	56.2	5.0	11.7	ATPB_BOVIN	ATP synthase subunit beta, mitochondrial precursor	−1.00	+1.73*	+1.72*
17	46.5	9.5	12.0	SERPH_BOVIN	Serpin H1	+1.35	−1.86**	−1.38
18	52.5	6.0	15.9	SBP1_BOVIN	Selenium-binding protein 1	−1.07	−1.92*	−2.06*
19	58.8	7.4	4.9	HRG_RABIT	Histidine-rich glycoprotein	−1.57	+1.76*	+1.12
20	25.1	6.0	14.3	PRDX6_BOVIN	Peroxiredoxin 6	+1.02	−1.59	−1.56*
21	25.1	6.0	27.2	PRDX6_BOVIN	Peroxiredoxin 6	+1.19	−1.50	−1.30*
22	25.1	6.0	18.8	PRDX6_BOVIN	Peroxiredoxin 6	+1.03	−1.54	−1.50**
23	26.7	6.5	18.9	TPIS_BOVIN	Triosephosphate isomerase	−1.10	−1.27	−1.39**
24	17.3	9.0	25.7	NDKB_BOVIN	Nucleoside diphosphate kinase B	−1.23	−1.55	−1.91*

1 L  =  low energy diet; M  =  medium energy diet, H  =  high energy diet.

2 Numbers correspond to the labelled spots in Figure 1.

3 Sequence coverage (%).

4 Accession number in NCBI (National Center for Biotechnology Information) and SwissProt database.

5 Fragment.

**different at *P*<0.01.

*different at *P*<0.05.

Further, the identified 12 proteins were categorized according to their known biological function based on UniProt database (http://www.uniprot.org/), literature, and the NCBI database. Accordingly, the comparison among gels pertaining to low and high energy groups indicated that expression of the transport protein transferrin was downregulated by 1.53 to 2.06 fold in response of high energy diet (Table 2). By contrast, feeding the low diet or increasing the energy intake from medium to high showed no significant effect on the expression of transferrin. Interestingly, expression profiles of proteins involved in cellular stress responses, such as heat shock cognate 71 kDa protein (HSP7C), albumin and PRDX6, or collagen-binding properties, like serpin H1, were downregulated in response to energy supply (Table 2). For example, data showed downregulation (by 1.26- and 1.31-fold) of HSP7C with increasing the amount of energy to medium and high, respectively, whereas the expression profiles of albumin and PRDX6 were downregulated only in response to an abrupt increase of energy from low to high. Also, the expression of serpin H1 or HSP47 was downregulated by 1.86 fold when dietary energy level was increased from medium to high, but not from low to medium. The expression of another protein involved in stress responses like protein disulfide-isomerase (PDIA1) was first upregulated 1.48 fold with increasing the energy level from low to medium, but downregulated (1.82 fold) in response to a further increase of energy to high level (Table 2). Expression of selenium-binding protein (SBP1) was also decreased with increasing energy level from medium to high (1.92 fold) and from low to high (2.06 fold), but not from low to medium (Table 2). In contrast, histidine-rich glycoprotein (HRG) was upregulated 1.76 fold when comparing low vs. medium diets only (Table 2).

The expression level of two proteins involved in metabolic functions such as retinal dehydrogenase 1 (AL1A1) and ATP synthase subunit beta, mitochondrial precursor (ATPB) were upregulated by roughly 1.6 to 1.7-fold with increasing the energy level in the diet (Table 2). In contrast, triosephosphate isomerase (TPI), a glycolytic enzyme, was downregulated by 1.39 fold in response to high energy supply compared to diet with a low supply of energy. The expression of nucleoside diphosphate kinase B (NDKB) was also downregulated 1.91 fold with increasing the energy in the diet from low to high (Table 2).

### Impact of Diet on Relative mRNA Expression

**Figure 2:**
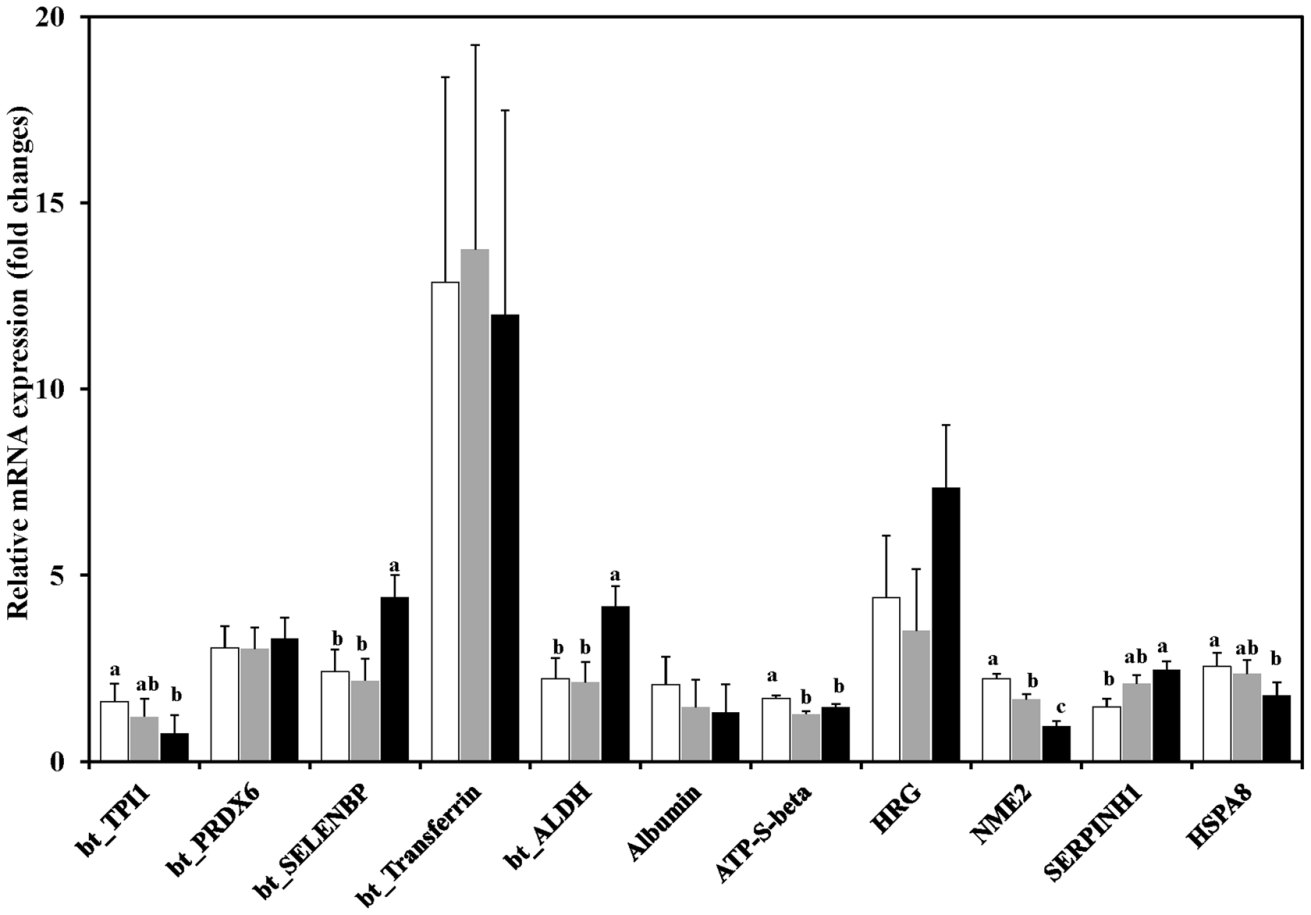
Relative mRNA expression of target genes in the rumen epithelium of goats. Animals were fed three different diets (white colour, low energy diet; gray color, medium energy diet; black colour, high energy diet). The mRNA expression of target genes was calculated relative to the expression of *ACTB* and *HPRT1*. Abbreviations of the genes are: HRG  =  Histidine-rich glycoprotein, NDK7 (NME2)  =  Nucleoside diphosphate kinase, HSPA8  =  Heat shock cognate 71 kDa, SERPINH1(HSP47)  =  Serpin H1, SELENBP1  =  Selenium-binding protein 1, TPI  =  Triosephosphate isomerise, ALDH1A  =  Aldehyde dehydrogenase1 family member A1, ATP5B  =  ATP synthase subunit beta, PRDX6  =  Peroxiredoxin 6, TF  =  Transferrin, Albumin  =  Albumin precursor, ACTB  =  β-actin, HPRT1  =  Hypoxanthine phosphoribosyltransferase. ^abc^ indicate difference at *P*<0.05.

Next to expression of proteins, we used quantitative RT-PCR analysis to evaluate whether regulation of selected proteins in response to energy feeding parallels mRNA expression of the corresponding genes (Figure 2). The relative expression of mRNA transcripts showed that genes encoding for metabolic functions like TPI (*bt_TPI1*), NDKB (*NME2*), ATPB (*ATP-S-beta*), or stress functions such as HSP7C (*HSPA8*) were downregulated, whereas those encoding for SBP1 (*bt_SELENBP*), AL1A1 (*bt_ALDH*), and serpin (*SERPINH1*) were upregulated with increasing the level of energy intake (Figure 2). In contrast, the expression of genes encoding for PRDX6 (*bt_PRDX6*), transferrin (*bt_Transferrin*), albumin precursor, and HRG did not respond to the dietary change (Figure 2).

## Discussion

Rumen epithelium is an important metabolic organ with high adaptive and proliferative properties, which, as the first line of defense, also has critical barrier functions in ruminants [5], [7]. There is a paucity of information regarding the regulation of protective factors of rumen epithelial cells, in particular, during stress conditions caused by high energy-induced acidotic insults. The more we know about the metabolic adaptive capacity and regulation of the protective functions of the rumen epithelium, the better we can shape our future feeding strategies for more efficient and healthy ruminant production. Therefore, this study sought to evaluate changes in the protein expression of rumen epithelium of goats in response to energy-rich diets. Herein, we identified downregulation of several proteins that are described to exert important cellular protective functions in stress situations in several other tissues, thus providing novel information regarding the damage process of protective epithelial function caused by acidotic challenge in the rumen. More specifically, we identified concomitant drops in the expression of HSP7C, serpin H1, albumin, PDIA1, SBP1, and PRDX6 in the gastrointestinal epithelial cells in response to high energy supply and the subsequent acidotic luminal stress. The study expands our previous article where we demonstrated an impairment of the barrier functions of rumen epithelium in an Ussing chamber experiment due to high energy supply and the resulting acidotic insult [4]. Whether the downregulation of HSP7C was directly involved in an increased permeability of rumen epithelium in response to energy-rich diet needs further clarification. Nonetheless, emerging evidence suggests that inhibition of this heat shock protein, even by a physiologically relevant increased temperature (i.e., from 37 to 41°C), produces a marked disruption in junctional localization of occludin protein during heat stress [22], thus contributing to the increase of intestinal permeability. Our data showed that feeding a high energy diet lowered rumen pH at 5.5, a value which is below the thresholds of subacute rumen acidosis [23], [24]. Besides acidotic pH values, subacute rumen acidosis is also characterized by high temperature, with ruminal temperatures exceeding 40°C [25], as well as the release of multiple toxic compounds [26]. Because occludin proteins also play a primary role as tight-cell junction structures of rumen epithelium, in particular in its stratum granulosum [7], it is reasonable to assume a potential involvement of inhibition of HSP7C expression in the impairment of the barrier functions of rumen epithelium reported in our previous article [4] and others [5], [7].

Understanding of the exact mechanism(s) behind downregulating effects of energy-rich diets on HSP7C expression would help in a better understanding in the disruption of epithelial barrier functions of the rumen due to such dietary conditions. Because the HSPA8 gene encoding for HSP7C also was downregulated in animals fed high energy diet, we believe that the mechanisms behind this downregulation are in the decreased transcription of this gene. In the rumen epithelium, HSP7C might enable epithelial cells to cope more easily with diurnal changes of fermentation patterns during rumen acidosis such as lowered pH and high osmotic pressure. Interestingly, our data fully agree with recent results that demonstrated a maximal expression of HSP70 at a pH range of 6.4–6.0, whereas a further decrease of mucosal pH to 5.8 caused the inhibition of this protein in that study [23]. The pH dependence of this protein indicates that pH might have played a role in downregulation of HSP7C, and also can explain downregulation of the other chaperone protein Serpin H1, a collagen-binding heat shock protein [28], which also binds to collagen by a pH-dependent manner, whereby this binding is abolished at a cellular pH of 6.3 [29]. Intracellular pH is higher than luminal pH but it is dependent on the latter variable [30], suggesting that goats of high energy diets also had lower intracellular pH. Downregulation of HSP7C and Serpin H1 upon the activity of acidotic challenge may suggest that these chaperone proteins are consumed in repairing sublethal cell damage without being adequately replenished, indicating that this kind of stress can jeopardize the synthesis of chaperone proteins in the rumen epithelial cells.

Our study also demonstrated downregulation of another chaperone protein like PDIA1 when shifting from low to high energy diet only. PDIA1 is a ubiquitously expressed oxidoreductase required for proper protein folding [31]. Increased oxidative stress is thought to modify PDIA1 at a critical cysteine residue involved in client protein binding, thereby downregulating the activity of native PDIA1 [32], [33].

We also observed that the expression of PRDX6, SBP1, and transferrin was downregulated in animals fed the high energy diet. While the downregulation of transferrin might be interpreted as a host reaction aiming to limit the access of potentially pathogenic bacteria to iron [34] that are induced by the acidotic insult [35], the downregulation of two other proteins involved in oxidative stress such as PRDX6 and SBP1 is not clear, but suggests their depletion in the rumen epithelium during a high energy-induced acidotic insult. PRDX6 is a member of the peroxiredoxin family of antioxidant enzymes, with bifunctional activities that has both glutathione peroxidase and phospholipase A2 enzymatic activities [36]. Earlier studies have suggested a mediator role of SBP1 in the intracellular transport of selenium [37], whereas clinical studies have revealed a possible mediator role of SBP1 for selenium's anticancer functions [38], [39]. This is the first time reporting SBP1 in the rumen epithelial cells. PRDX6 expression in rumen epithelium has been reported previously, whereby its expression was downregulated, in the same manner as in our study, in response to energy-rich feeding in sheep [8]. A depletion of PRDX6 is known to decrease the host ability to counteract bacterial infections in human epithelial cells [40], suggesting that a depletion of PRDX6 in response to energy-rich feeding might be a risk factor for local inflammation and opening the port of entry of infectious agents into systemic circulation. Rumen acidosis has been often associated with local inflammation, ruminitis, and increased apoptosis [41], and this might also explain upregulation of HRG in the rumen epithelium of goats in response to energy supply. Recent data suggest that HRG can aid the phagocytosis and removal of necrotic cells [42].

In contrast, other results of this study showed upregulation of proteins involved in metabolic functions such as AL1A1 and ATPB. The role of retinal dehydrogenase 1 is to generate retinoic acid (RA), which is needed for the differentiation of specific gut epithelial cells [43], [44]. To our knowledge, this is the first study to report AL1A1 expression in the rumen epithelium. In fact, if we hypothesize that RA could be important for the differentiation and proliferation of rumen epithelial cells, we could conclude that upregulation of AL1A1 expression upon the effects of dietary energy supply was part of the host response because these cells require greater quantities of RA due to their faster growth and differentiation rate in response to luminal short chain fatty acids [7], [12]. On the other hand, RA is known to play a role in the repair of the intestinal epithelium [45] and maintaining its barrier function [46], suggesting that the increase of AL1A1 expression might also be interpreted as an effort of the host to counteract the negative effects of acidotic insult on barrier functions.

Upregulation of ATPB in the epithelium of goats in response to energy supply supports the findings of Bondzio et al. [8], who have identified ATP5G (ATP synthase subunit gamma) to be up-regulated in sheep fed a concentrate supplemented diet, and suggests an increased energy requirement during enhanced proliferation of germinative epithelial cell layers of the rumen [47] or an increase of electrochemical gradients in response to energy supply [12]. On the other hand, the study demonstrated downregulation of other two enzymes involved in energy metabolism such as NDKB and TPI in response to high energy supply. Downregulation of NDKB can be attributed to higher luminal glucose in response to energy-rich diet [2], [27], as a chronic exposure of glucose has been shown to inhibit NDPK activity [48]. On the other hand, lowered levels of TPI, an essential glycolytic enzyme, may suggest redirection of energy fluxes in rumen epithelial cells, probably in the form of NADPH, observed during acidotic-induced stresses [49].

Interestingly, the results of the qPCR analysis demonstrated downregulation in the expression of mRNA transcripts of *bt_TPI1* and *NME2* as well as upregulation of *bt_ALDH*. These findings suggest that differences observed for TPI and NDKB as well as AL1A1, in response to high energy supply, are controlled at transcriptional levels. These findings also indicate a good concordance between mRNA and protein changes for these proteins with important metabolic function. A good agreement was also observed between mRNA transcripts and expression of proteins with stress protective and other functions such as HSP7C and albumin. However, the expression profiles of other proteins that also involved in stress or other cellular functions were different from the corresponding mRNA expression such as *bt_PRDX6*, *bt_Transferrin*, *SERPINH1* and HRG, indicating that these changes were not controlled at transcriptional level.

In summary, decreasing expression of chaperone proteins in the rumen epithelial cells in response to high energy supply may suggest that these cells were less well protected against the potentially harmful content of the ruminal lumen, which might have consequences for rumen and systemic health. Our findings also indicate that energy-rich diets and the resulting acidotic insult may render rumen epithelial cells more vulnerable to cellular damage and apoptosis by attenuating their cell defense system. Thus, the findings of the current study and of our previous study [4] support the contention that the increase of permeability of rumen epithelium in response to feeding of energy-rich diets is initiated by proteomic changes related to impairment of cellular protection system, brought about by these feeding practices.

## Supporting Information

Table S1 (DOC)
