# Supplementary material for: Downregulation of Cellular Protective Factors of Rumen Epithelium in Goats Fed High Energy Diet
Source: PLoS One. 2013 Dec 9;8(12):e81602. doi: 10.1371/journal.pone.0081602 (PMC3857193; doi:10.1371/journal.pone.0081602)
Supplement: Table S1 — (DOC) [file pone.0081602.s001.doc]

**SUPPLEMENTARY MATERIAL Table 1** Primers used for quantitative PCR

| Gene | Gene name | Sequence |  | Amplicon  (bp) | Accession No.^3^ | Efficiency  (%) | R^2^ |
| --- | --- | --- | --- | --- | --- | --- | --- |
| HRG | Histidine-rich glycoprotein | TGTTCTATGATGTAGAAGCCTCTGAC | S^1^ | 111 | NM_173919 | 96.34 | 0.997 |
|  |  | AATGATGTTGCACAGCACTGAAG | AS^2^ |  |  |  |  |
| NDK7 (NME2) | Nucleoside diphosphate kinase | GTCTGGGAGGGCCTGAATGT | S | 74 | NM_001015656 | 94.16 | 0.991 |
|  |  | GGCTTAGAATCTGCTGGGTTG | AS |  |  |  |  |
| HSPA8 | Heat shock cognate 71 kDa | CAAACAGAAGATTATTGACAAGCGTAA | S | 89 | XR_139519 | 82.410 | 0.990 |
|  |  | GCTGATGTTCAAATTCTTCCTTCTCT | AS |  |  |  |  |
| SERPINH1(HSP47) | Serpin H1 | ACCCACGACTTACAGAAACACTTG | S | 87 | NM_001046063 | 104.240 | 0.998 |
|  |  | GCCCGACATGCGAGACA | AS |  |  |  |  |
| SELENBP1 | Selenium-binding protein 1 | ATCTCATCAGGGAAGGCTCTGTG | S | 93 | NM_001046048 | 94.927 | 0.992 |
|  |  | CAAAATCCACTAGGAAGTTGGGG | AS |  |  |  |  |
| TPI | Triosephosphate isomerase | GAAGATGAACGGGAGGAAGAACA | S | 143 | BT021064 | 94.893 | 0.994 |
|  |  | CAATCTTGGGATCTAGCTTCTGCC | AS |  |  |  |  |
| ALDH1A | Aldehyde dehydrogenase1 family member A1 | TGTGTGGCCAAATCATTCCTT | S | 88 | NM_174239 | 98.798 | 0.996 |
|  |  | ACCACTGTGTTTCCGCAGCT | AS |  |  |  |  |
| ATP5B | ATP synthase subunit beta | GCTGAGGTCTTTACTGGTCATTTG | S | 95 | X05605 | 95.488 | 0.997 |
|  |  | AGATGGTCATATTCACCTGCCAA | AS |  |  |  |  |
| PRDX6 | Peroxiredoxin 6 | TGGACCCAGCAGAGAAAGATG | S | 108 | NM_174643 | 91.555 | 0.994 |
|  |  | TGGTAGCTGGGTAGAGGATGGA | AS |  |  |  |  |
| TF | Transferrin | GCATTGGCTCAGAGAAGGGTACA | S | 130 | NM_177484 | 90.300 | 0.994 |
|  |  | ACAGTCTGGTCCTTCACAAAGGC | AS |  |  |  |  |
| Albumin | Albumin precursor | GCGACCTACTTGAATGCGC | S | 108 | EU746506 | 98.520 | 0.999 |
|  |  | ACACAGGCTTATCACAGCATTCC | AS |  |  |  |  |
| ACTB | β-actin | GGCCAACCGTGAGAAGATGA | S | 85 | U39357 | 97.712 | 0.999 |
|  |  | GGACAGCACAGCCTGGATG | AS |  |  |  |  |
| HPRT1 | Hypoxanthine phosphoribosyltransferase | CTGTGGATTTTATCAGACTGAAGAGC | S | 89 | NM_001034035 | 98.529 | 0.997 |
|  |  | TAAAGTTGAGAGATCATCTCCACCAA | AS |  |  |  |  |

^1^Sense (S)

^2^Antisense (AS)

^3^National Center for Biotechnology Information (NCBI) Entrez Gene (<http://www.ncbi.nlm.nih.gov/sites/entrez?db=gene>).
